# A Novel Bradykinin-Related Peptide, RVA-Thr^6^-BK, from the Skin Secretion of the Hejiang Frog; *Ordorrana hejiangensis*: Effects of Mammalian Isolated Smooth Muscle

**DOI:** 10.3390/toxins11070376

**Published:** 2019-06-28

**Authors:** Yue Wu, Daning Shi, Xiaoling Chen, Lei Wang, Yuan Ying, Chengbang Ma, Xinping Xi, Mei Zhou, Tianbao Chen, Chris Shaw

**Affiliations:** 1 Natural Drug Discovery Group, School of Pharmacy, Queen’s University Belfast, Belfast BT9 7BL, Northern Ireland, UK; 2 School of Government, Peking University, No 114, The Leo KoGuan Building, Beijing 100871, China

**Keywords:** bradykinin-related peptide (BRP), RVA-Thr^6^-BK, site-substitution variant, agonist, antagonist, myotropic actions

## Abstract

A novel naturally-occurring bradykinin-related peptide (BRP) with an N-terminal extension, named RVA-Thr^6^-Bradykinin (RVA-Thr^6^-BK), was here isolated and identified from the cutaneous secretion of *Odorrana hejiangensis (O. hejiangensis)*. Thereafter, in order to evaluate the difference in myotropic actions, a leucine site-substitution variant from *Amolops wuyiensis* skin secretion, RVA-Leu^1^, Thr^6^-BK, was chemically synthesized. Myotropic studies indicated that single-site arginine (R) replacement by leucine (L) at position-4 from the N-terminus, altered the action of RVA-Thr^6^-BK from an agonist to an antagonist of BK actions on rat ileum smooth muscle. Additionally, both BK N-terminal extended derivatives (RVA-Thr^6^-BK and RVA-Leu^1^, Thr^6^-BK) exerted identical myotropic actions to BK, such as increasing the frequency of contraction, contracting and relaxing the rat uterus, bladder and artery preparations, respectively.

## 1. Introduction

Bradykinin (BK) is ubiquitous in mammals and its generation from precursor kininogens by plasma/tissue kallikreins in the human kallikrein-kinin system (KKS), has been well-researched [1,2,3]. Currently, BK has been proved to be a critical participant associated with human physiological and pathological processes, such as adjusting the functions of the cardiovascular, renal and nervous systems, smooth muscle contraction, glucose metabolism, cell proliferation, inflammation and the production of pain [3,4]. In contrast to mammalian KKS, there is no compelling evidence of the existence of KKS among anura [5,6,7]. Surprisingly, following the initial discovery mammalian BK from *Rana temporaria* in 1965, numerous identical BK and its structural analogues with potent smooth muscle stimulant have been found in anuran amphibian venom apparatus-skin [7,8,9,10,11,12]. Moreover, an extraordinary structural diversity was observed in anuran skin bradykinin-related peptides (BRPs). In addition to single/multiple site-substitution that frequently occurred at Arg^1^ and Ser^6^, there is some deficiency of a certain amino-acid residue, such as the deletion of arginine at N-/C- terminus (des-Arg^9^ and des-Arg^1^) [9,13,14,15]. On the other hand, hydroxylation of the proline residue often appeared among anuran BRPs, typical example like Hyp^3^-BK successively isolated from three species *Rana temporaria*, *Phyllomedusa azurea* and *Hylarana guentheri* [9,14,16]. In comparison of original BK, its structural isoforms, in general, displayed N-terminal extension and/or C-terminal extension, as well as segment insertion [9,17]. These variables regularly happened in multiples, ultimately conferring BPRs structural diversity [9,12]. However, the reasons for their diverse expression patterns and physiological roles of venom BK and BRPs in non-mammalian vertebrate, of the many the anuran skin BRPs, are not clear yet. 

A naturally-occurring leucine-substituted BK analogue, RVA-Leu^1^, Thr^6^-BK, also known as amolopkinin W1, was identified in *Amolops wuyiensis* skin secretion and has been reported as an antagonist in inhibiting contractility of BK on rat isolated ileum [18]. In this study, a novel BK structural variant was identified from *O. hejiangensis*. It is the first BRP from the defensive skin secretion in this frog species. This peptide primary structure, RVA-Thr^6^-BK, share high sequence similarity to RVA-Leu^1^, Thr^6^-BK initially found able to antagonize the relaxant effect observed following BK application to a rat arterial smooth muscle preparation in vitro [18]. Their corresponding chemically synthesized replicate was subjected to our myotropic activity evaluation on rat isolated vascular and non-vascular smooth muscles respectively.

## 2. Results

### 2.1. Molecular Cloning of Biosynthetic Precursor-Encoding cDNA and Structural Characterization of RVA-Thr^6^-BK from RP-HPLC Fractions of Skin Secretion

**Figure 1:**
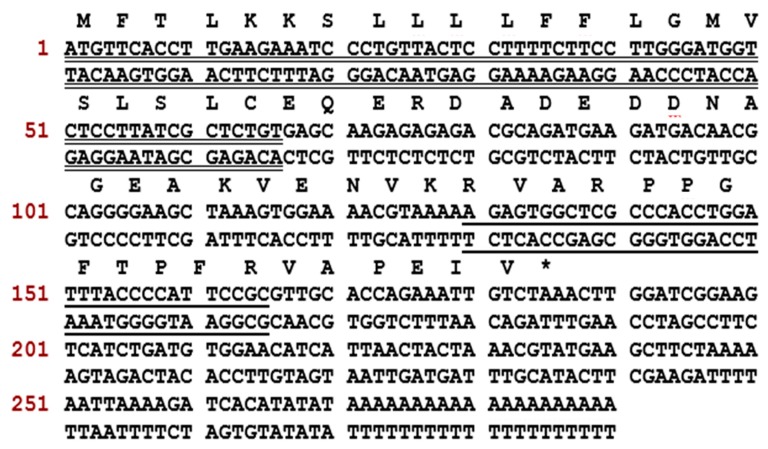
Nucleotide and translated open-reading frame amino-acid sequence of cDNA encoding the RVA-Thr^6^-BK precursor cloned from a skin secretion library of *O. hejiangensis.* The putative signal peptide and mature peptide are labelled by double-underlining and single-underlining, respectively. The asterisk indicates the stop codon.

**Figure 2:**
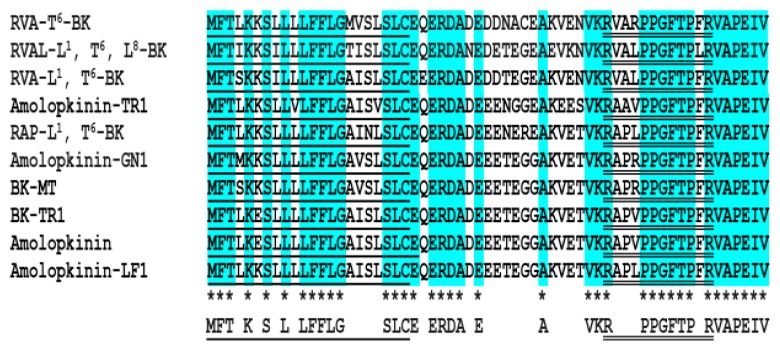
Amino-acid sequence alignment of RVA-Thr^6^-BK precursor and other BRP precursors from the skin secretion of several frog species. These data were obtained from GenBank and their corresponding accession numbers from top to bottom are CDO58214, CAR82590, ADV36199, SBN54116, ADM34221, ADM34238, ABF74734, ADV36198 and ADM34255, respectively. Single- and double-underlining represent signal and mature peptide amino-acid sequences, respectively. Asterisks indicate consensus residues.

A full-length biosynthetic BRP-precursor cDNA was consistently cloned from the cDNA library constructed from the skin secretion of *O. hejiangensis* (Figure 1). Similarly, in comparison of nine known BRP cDNA transcripts with the same length (Figure 2), this skin-kininogen precursor had 61 amino-acid residues encoding a single 12-mer BK-like peptide following a C-terminal extension sequence VAPEIV. The typical signal peptide consisting of 22 amino-acid residues in the translated open-reading frame was identified through NCBI-BLAST, whereas the convertase processing site, lysine, is not the most common. In accordance with the presence of a segment with 3 amino-acid residues (RVA) at the N-terminal and a threonine residue at position 6 in the BK-like sequence, the novel BK-like was named RVA-Thr^6^-BK. The RVA-Thr^6^-BK biosynthetic precursor-encoding cDNA has been deposited in the NCBI Database (accession code; MK863068).

### 2.2. Isolation and Structural Characterization of RVA-Thr^6^-BK from RP-HPLC Fractions of Skin Secretion

**Figure 3:**
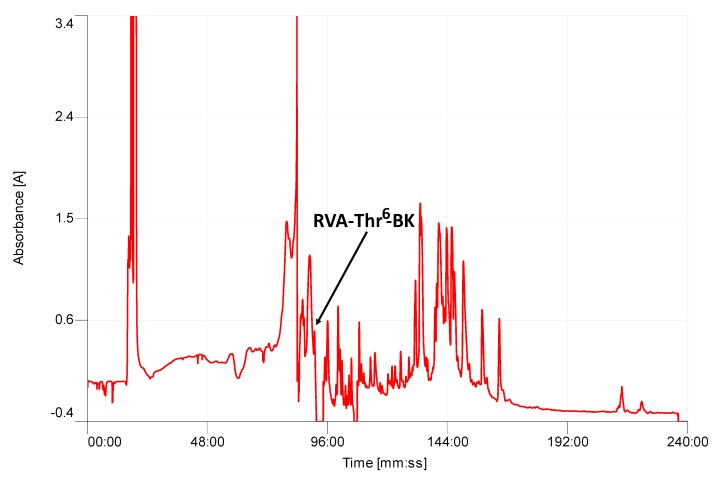
Region of RP-HPLC chromatogram of *O. hejiangensis* skin secretion indicating the retention time of RVA-Thr^6^-BK. Absorbance wavelength was set at 214 nm.

The average molecular mass of the putative mature peptide from the cloned precursor was calculated as 1400.64 Da. Thereafter, the presence of mature RVA-Thr^6^-BK with 12 amino-acids, was further confirmed by RP-HPLC isolation from within the secretion (Figure 3) and tandem mass spectrometry (MS/MS) fragmentation sequencing (Table 1), with the retention time of 89 min.

### 2.3. Myotropic Activities of RVA-Thr^6^-BK and RVA-Leu^1^, Thr^6^-BK

**Figure 4:**
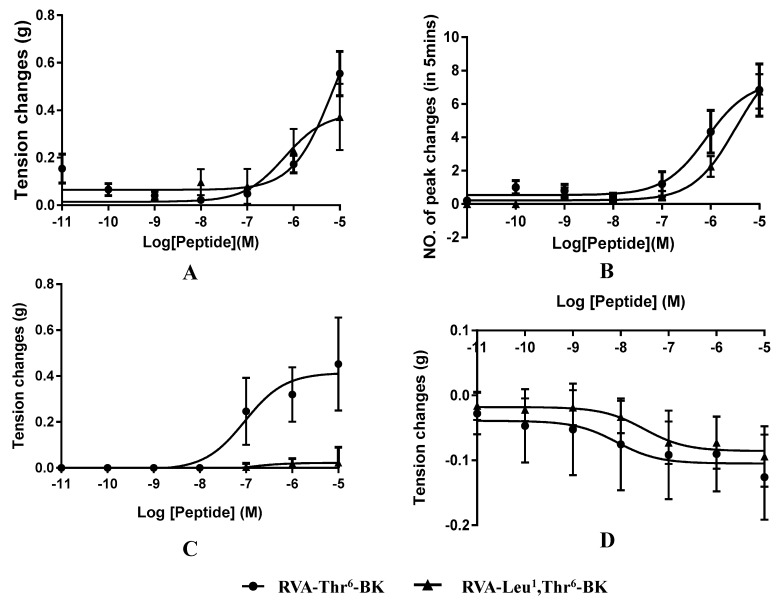
Dose–response curve of RVA-Thr^6^-BK (●) and RVA-Leu^1^, Thr^6^-BK (▴) using (**A**) rat bladder smooth muscle preparations (N = 4, *n* = 8), (N refers to the number of animals being study, n refers to the number of tests conducted), (**B**) rat uterus smooth muscle preparations (N = 4, *n* = 7), (**C**) rat ileum smooth muscle preparations (N = 3, *n* = 4) and (**D**) rat tail artery smooth muscle preparations (N = 4, *n* = 8), respectively. Data indicate tension changes compared with non-peptide exposed controls.

Myotropic activities of RVA-Thr^6^-BK and RVA-Leu^1^, Thr^6^-BK were investigated in four different types of rat smooth muscle preparations (Table 2). These two peptides displayed BK-like contractile effects on isolated bladder preparations (rat urinary) (Figure 4A) and were found to increase the frequency of contraction of the uterus (Figure 4B). Moreover, at a higher concentration (10 µM), RVA-Thr^6^-BK caused greater contraction of bladder preparations than its leucine-substituted isoform; however, it required higher threshold concentration of RVA-Thr^6^-BK to produce a constriction response (Table 2 and Figure 4B). Interestingly, RVA-Thr^6^-BK was capable of causing potent contraction of rat ileum preparations, whereas, there was no significant response from rat ileum smooth muscle with RVA-Leu^1^, Thr^6^-BK (Figure 4C). In contrast to the contractility of the non-vascular isolated smooth muscle of rat, both BK structural variants used here demonstrated relaxing action on artery preparations of the rat (Figure 4D). 

### 2.4. Assessment of Cytotoxic Effect on Mammalian Erythrocytes

**Figure 5:**
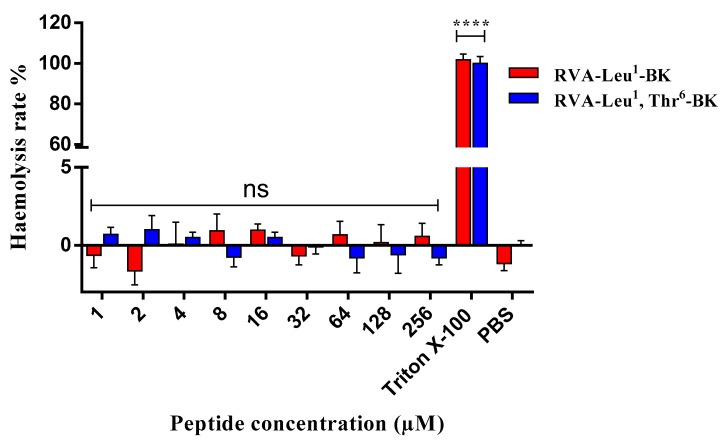
Haemolytic activities of RVA-Thr^6^-BK (red) and RVA-Leu^1^, Thr^6^-BK (blue) using mammalian erythrocytes. **** *p* < 0.0001; ns; no significance.

To evaluate the potential toxicity of RVA-Thr^6^-BK and its analogue, RVA-Leu^1^, Thr^6^-BK, the healthy mammalian red blood cells were treated with these two peptides (Figure 5). The results indicated that both peptides showed no haemolytic activity in vitro. 

## 3. Discussion

Some efforts have been made in anuran amphibian skin research and conservation over several decades [11,12,19,20]. As a consequence of these investigations, an increasing number of biologically-active peptides (frequently anti-microorganism peptides but also neuroactive peptides and protease inhibitor peptides) with a wide range of functions have been discovered [11,12,19,20,21]. Among these, it is revealed that defensive neuroactive peptides with myotropic activity are remarkably varied in anuran skin secretions, and these small molecule peptides can evoke contractions and relaxations on smooth muscles, BK and BRPs being a typical example [7,8,9,12]. Herein, a novel BK-like peptide and its corresponding precursor-encoding cDNA were identified. It was found that RVA-Thr^6^-BK precursor has a highly conserved structure consisting of an N-terminal signal peptide domain followed by an acidic amino-acid-rich (spacer) domain and terminating in a single mature BK-like peptide coding domain (Figure 2). This result is similar to some of the BRP precursors in other frog species [7,14,18]. In contrast, there are only two types of BK peptides found in human (BK and Lys^0^-BK). Conlon and Yano proved that there are no blood-derived kinins in the frog plasma after treatment with trypsin [11]. Another convincing piece of evidence of no detectable kininogens in toad plasma was that many amphibians express BK and BRPs by processing skin-kininogens/preprobradykinins in their elaborate skin kinin system, and such a producing site differs from that of human KKS [5]. Some identical BK derivatives found in anuran skin were also identified in other non-mammalian vertebrate species, such as Arg^0^, Trp^5^, Leu^8^-BK and Thr^6^, Leu^8^-BK [21,22]. Therefore, it has been proposed that non-mammalian vertebrates develop similar BK and BRPs for their defensive systems, instead of having physiological roles as in mammals [7,11]. However, it is still a point of contention whether the amphibian skin-kininogens/preprobradykinins and plasma-kininogens of humans, are indeed expressed by homologous genes.

In this investigation, RVA-Thr^6^-BK was shown to have the opposite effect of RVA-Leu^1^, Thr^6^-BK on isolated rat ileum smooth muscle in vitro, where RVA-Leu^1^, Thr^6^-BK had no direct myotropic effects on rat ileum smooth muscle, which is consistent with our previous study [18]. Moreover, another homologous analogue RAA-Leu^1^, Thr^6^-BK also found in *Amolops wuyiensis* skin secretion at the same time was also reported as an inhibitor of BK-induced contraction of isolated rat ileum smooth muscle [18], indicating that the Val^2^ amino-acid replacement is less likely to result in the alteration of myotropic action among BRPs. The difference between of RVA-Thr^6^-BK and RVA-Leu^1^, Thr^6^-BK solely involves arginine/leucine site-substitution at position 4 from the N-terminus, suggesting this is the core site for converting RVA-Thr^6^-BK from an agonist to an antagonist of BK on rat ileum smooth muscle. Besides, BK-like vasodilator action, as well as contraction effects on bladder and uterus preparations of RVA-Leu^1^, Thr^6^-BK, are first described here. It was shown that the biological effects of BK are mediated by specific receptors, the B_1_ and B_2_ receptors which are classified as G-protein-coupled receptors having seven transmembrane domains crossing the lipid bilayer, three extracellular and intracellular loops, an exposed N-terminal domain and a cytoplasmic C-terminal domain [4,23,24,25]. Previous literature demonstrated that the conformation of BK was oriented randomly in aqueous environments, but nevertheless, a specific segment in the BK sequence from residues 2–5, PPGF, was shown to have a preference to form a β-turn conformation [26,27]. However, the unambiguous bioactive conformation for BK binding to its receptor remains inconclusive.

In the haemolytic test, the haemoglobin release in erythrocytes was not observed in both myotropic peptides. However, haemolytic activity in vitro is the initial test for evaluating toxic potential and this assay is not sufficient to completely exclude these peptides with the toxicity. Further investigation on cytotoxicity in more complex experimental models, such as cell lines [28] and insect models (e.g., *Galleria mellonella* larva) [29], need to be taken into account to ensure the development of safe for pharmaceutical use. 

In summary, a novel BK analogue is described here which has not been reported previously and this is also the first BRP isolated from *O. hejiangensis*. Both arginine/leucine site-substitution in BK N-terminal extension analogues displayed similar myotropic properties to BK, except that Leu-4 site-substitution produced an antagonist of BK on the rat ileum. Our discovery and exploration of the myotropic activities of anuran skin BRPs provide unique opportunities to better understand the key role of venom BK and BRPs in non-mammalian vertebrates. Additionally, the structural diversity and BK-functional-related actions suggest that anuran amphibians BRPs are unique tools for studying structure-activity relationships as well as developing new or even more potent drugs with therapeutic potential. 

## 4. Materials and Methods

### 4.1. Skin Secretion Acquisition

Specimens of *O. hejiangensis* (n = 8) of undetermined sex were obtained in China and were kept in a purpose-designed amphibian facility prior to secretion harvesting. The skin secretions were harvested using gentle transdermal electrical stimulation initially described by Tyler and co-workers in 1992 [30]. Briefly, following stimulation by platinum electrodes (6V, 4ms pulse-width, 50 Hz, 20s duration), the secretions from the dorsal skin of frogs were washed with deionised water, snap-frozen in liquid nitrogen and lyophilised. The lyophilised sample was stored at −20 °C before analysis. This study was performed under the UK Animal (Scientific Procedures) Act 1986, project license PPL 2694, issued by the Department of Health, Social Services and Public Safety, Northern Ireland. The procedures had been overseen by the IACUC of Queen’s University Belfast, and approved on 1 March 2011. 

### 4.2. “Shotgun” Cloning of cDNA Encoding RVA-Thr^6^-BK Biosynthetic Precursor from Skin Secretion

Polyadenylated mRNA was extracted from 5 mg of lyophilised *O. hejiangensis* skin secretion using magnetic oligo-dT beads (Dynal Biotech, Bromborough, UK) and then subjected to 3′- and 5′-RACE procedures to obtain a full-length cDNA employing a 3′-/5′-SMART-RACE kit (Clontech, Mountain View, CA, USA) essentially as described previously [31]. Briefly, the 3′-RACE reactions utilized a Nested Universal Primer A (NUP) (provided with the kit) and a degenerate sense primer (S1: 5′-CCRVCNGGGTTYASSCCWTTY-3′) that was designed to a nucleotide sequence encoding the common internal sequence of anuran skin-derived BRPs, Pro-Pro-Gly-Phe-X-Pro-Phe, where X is either serine or threonine. Thereafter, PCR products were gel-purified, cloned using a pGEM-T vector system (Promega Corporation, Masison, WI, USA) and sequenced using an ABI 3100 automated capillary sequencer. Accordingly, the 5′-RACE reactions employed an antisense primer (S2: 5′-CATCAGATGACTGCCGATCCAA-3′), which was designed to a domain located within the 3′ non-translated region of the obtained 3′-RACE transcripts, and a Universal Primer A Mix (UPM) (supplied with the kit). Similarly, 5’-RACE PCR products were gel-purified, cloned using a pGEM-T vector system and finally sequenced. 

### 4.3. Isolation and Structural Characterization of RVA-Thr^6^-BK from Skin Secretion 

The isolation and structural characterization of RVA-Thr^6^-BK from the lyophilised *O. hejiangensis* skin secretion was carried out as previous study [32]. Briefly, a Cecil CE4200 Adept (Cambridge, UK) gradient reverse-phase High Performance Liquid Chromatography (RP-HPLC) system fitted with a column (Phenomenex C-5, 0.46 cm × 25 cm) was employed to isolate the skin peptides from 5 mg of lyophilise skin secretion. The fractions were collected automatically at 1 min intervals. Thereafter, each fraction was analyzed using MALDI-TOF MS (Perseptive Biosystems, Foster City, MA, USA) and an LCQ-Fleet electrospray ion-trap mass spectrometer (Thermo Fisher Scientific, San Jose, CA, USA) for MS/MS fragmentation sequencing. The primary structure of the novel peptide, RVA-Thr^6^-BK, was thus established.

### 4.4. RVA-Thr^6^-BK and Its Site-Substituted Analogue, RVA-Leu^1^, Thr^6^-BK: Synthesis and Purification

Here, the peptide, RVA-Leu^1^, Thr^6^-BK (RVALPPGFTPFR), was synthesized for investigating its myotropic activity directly in comparison with that of RVA-Thr^6^-BK (RVARPPGFTPFR). Sufficient quantities to detect the bioactivities of each peptide were obtained using the Tribute peptide synthesizer (Protein Technologies, Tucson, AZ, USA) along with standard Fmoc-chemistry according to our previous study [32].

### 4.5. Myotropic Activity Evaluation on Smooth Muscles

Following asphyxiation (by CO_2_) and cervical dislocation, healthy female Wistar rats (250–350 g) were dissected from the mid-ventral line and these procedures were performed as required by UK Animal Research guidelines. After cutting the intact isolated organs into the desired sizes (10mm-length and 5mm-width parallel muscle bundles for bladder, 5 mm-width rings for uterine horn, 5 mm-length rings for ileum and 2 mm-width rings for tail artery), each strip hooked on tail-to-head was linked with fine silk ligatures (0.2 mm), respectively, and mounted into 2 mL organ baths with transducers. Meanwhile, to mimic the internal physiological environment, organ baths were filled with Kreb’s solution at 37 °C flowing through the bath at 2 mL/min with constant bubbling (95% O_2_, 5% CO_2_). After 20 min of a resting period without drugs, tissues were incubated with drugs from 10^−11^ to 10^−5^ M and the tension changes were recorded via pressure transducers with a PowerLab System (AD Instruments Pty Ltd., Bella Vista, NSW, Australia). The retention periods for each peptide concentration were at least 5 min. 

### 4.6. Haemolysis Assay 

Haemoglobin release in the horse erythrocytes (2% solution) (TCS Biosciences Ltd., Buckingham, UK) were determined to evaluate the potential toxicity of RVA-Thr^6^-BK and its site-substituted analogue, which was described previously [32]. The horse erythrocytes were treated with each peptide at the intended concentrations (i.e., from 1 to 256 μM) for 2 h at 37 °C. 1% Triton X-100 and phosphate-buffered saline (PBS) were served as the negative and positive controls, respectively. The supernatant of each tube was obtained by centrifugation at 1000× *g* for 5 min and the OD value of supernatant was analyzed at a wavelength of 550 nm using the plate reader.

### 4.7. Statistical Analysis

Data were analyzed to obtain the mean and standard error of responses by Student’s test and dose–response curves were constructed using a best-fit algorithm through the data analysis package Prism 6 (GraphPad Software, La Jolla, CA, USA). EC50 values were calculated from the normalized curves.

## Figures and Tables

**Table 1:** Calculated singly-charged and doubly-charged b-ions and y-ions produced by MS/MS fragmentation of RVA-Thr^6^-BK identified in skin secretion. Observed ions following MS/MS fragmentation are indicated in red and blue typefaces.

#1	b(1+)	b(2+)	b(3+)	Seq.	y(1+)	y(2+)	y(3+)	#2
1	157.10840	79.05784	53.04098	R				12
2	256.17682	128.59205	86.06379	V	1244.68992	622.84860	415.56816	11
3	327.21394	164.11061	109.74283	A	1145.62150	573.31439	382.54535	10
4	483.31506	242.16117	161.77654	R	1074.58438	537.79583	358.86631	9
5	580.36783	290.68755	194.12746	P	918.48326	459.74527	306.83260	8
6	677.42060	339.21394	226.47838	P	821.43049	411.21888	274.48168	7
7	734.44207	367.72467	245.48554	G	724.37772	362.69250	242.13076	6
8	881.51049	441.25888	294.50835	F	667.35625	334.18176	223.12360	5
9	982.55817	491.78272	328.19091	T	520.28783	260.64755	174.10079	4
10	1079.61094	540.30911	360.54183	P	419.24015	210.12371	140.41823	3
11	1226.67936	613.84332	409.56464	F	322.18738	161.59733	108.06731	2
12				R	175.11896	88.06312	59.04450	1

**Table 2:** Myotropic activities of RVA-Thr^6^-BK and RVA-Leu^1^, Thr^6^-BK in four different types of rat smooth muscle preparations. E max indicates the maximum response of isolated tissue inducing by BRPs at a peptide concentration of 10^−5^M. TC and EC_50_ represent threshold concentration and the concentration of BRPs giving the half-maximal response respectively. N means undetectable value under 10^−5^M of BRPs.

Isolated Tissue	E Max	TC (nM)	EC50 (nM)
	RVA-Thr^6^-BK/RVA-Leu^1^, Thr^6^-BK		
Bladder	0.56/0.38 (g)	100.00/10.00	7102.00/636.70
Uterus	7.00/7.00 (peaks/5 min)	10.00/100.00	817.20/3270.00
Ileum	0.43/N (g)	1.00/N	93.70/N
Tail artery	−0.13/−0.08 (g)	1.00/1	8.28/30.91
